# Characterization of *Aspergillus* section *Flavi* associated with stored grains

**DOI:** 10.1007/s12550-023-00514-1

**Published:** 2024-01-17

**Authors:** Eman G. A. M. El-Dawy, Youssuf A. Gherbawy, Mohamed A. Hussein

**Affiliations:** 1 Botany and Microbiology Department, Faculty of Science, South Valley University, Qena, Egypt; 2 Applied and Environmental Microbiology Center, South Valley University, Qena, Egypt

**Keywords:** *Flavi*, Calmodulin gene, Aflatoxins, *Trichoderma*

## Abstract

Increased frequencies of *Aspergillus* section *Flavi* and aflatoxins in cereal grains have been seen in recent years due to changes in climate circumstances, such as high temperatures and drought. To assess the microbiological risks of contamination, it is critical to have a reliable and accurate means of identifying the fungi. The main goal of this study was to characterize *Aspergillus* species from section *Flavi* obtained from twenty-three samples of barley and maize grains, gathered from different markets in Qena, Egypt, using morphological and molecular techniques. Twenty-three isolates were chosen, one isolate from each sample; they were identified as *A. aflatoxiformans* (4 isolates), *A. flavus* (18), and *A. parasiticus* (1)*.* The existence of four aflatoxin biosynthesis genes was also investigated in relation to the strains’ ability to produce total aflatoxins and aflatoxin B1, focusing on the regulatory gene *aflR* and the structural genes *aflD* and *aflM*. All strains producing aflatoxins were linked to the presence of *aflR1* and/or *aflR2*, except two isolates that exhibited aflatoxins but from which *aflR1* or *aflR2* were not detected, which may be due to one or more missing or unstudied additional genes involved in aflatoxin production. *AflD* and *aflM* genes were amplified by 10 and 9 isolates, respectively. Five samples of barley and maize were contaminated by aflatoxins. Fifteen isolates were positive for producing total aflatoxins in the range of 0.1–240 ppm. Antagonistic activity of *Trichoderma viride* against *A. flavus* (F5) was assessed at 31.3%. *Trichoderma* reduced total aflatoxins in all treated seeds, particularly those subjected to *Trichoderma* formulation.

## Introduction

Cereal crops are susceptible to fungal development, which can lead to the synthesis of diverse secondary metabolites throughout their growing, harvesting, storage, and processing stages (Tebele et al. 2020; Abdel-Nasser et al. 2022). The proliferation of fungi that are mycotoxigenic on cereals in the Middle East and Africa regions is facilitated by prevailing environmental factors, including high temperatures, excessive humidity, erratic rainfall patterns, and recurrent periods of drought (Tebele et al. 2020). *Aspergillus* and *Penicillium* species, particularly *Aspergillus flavus*, commonly proliferate on essential commodities such as maize, peanuts, tree nuts, spices, and cotton seed, as well as barley, during storage conditions (Yu and Pedroso 2023).

Six economically significant species of *Aspergillus* section *Flavi*, which are closely related morphologically and phylogenetically, are commonly separated into two categories based on their effects on food or human health. The first group consists of *Aspergillus flavus*, *A. parasiticus*, and *A. nomius*, which can damage nuts, spices, and peanuts as well as grains such as wheat and rye in storage conditions (Rigo et al. 2002; Hedayati et al. 2007). Furthermore, these species can produce carcinogenic secondary metabolites, the aflatoxins (Kurtzman et al. 1987; Yuan et al. 1995; Samson et al. 2000; Hedayati et al. 2007; Godet and Munau 2010). Often, several species of *Aspergillus* section *Flavi* are frequently misidentified and named as *A. flavus*. There is observed variability in the phenotype of *A. flavus*, which includes differences in sclerotia development, culture properties, and aflatoxin production capacity. The observed variability among the isolates of *A. flavus* indicates the necessity for a more comprehensive taxonomic classification (Okoth et al. 2018). Other species are included in this group, but these species are rarely isolated. *Aspergillus bombycis* was characterized by Peterson et al. (2001) using nine isolates that were obtained from families that raised silkworms. Frisvad et al. (2005) elevated a variety of *A. flavus* and *A. flavus* var*. parvisclerotigenus*, to species level as *Aspergillus parvisclerotigenus*. Pildain et al. (2008) described *Aspergillus arachidicola* and *Aspergillus minisclerotigenes*. *A. arachidicola* was isolated in Argentina from *Arachis*. Geiser et al. (2000) classified several strains of *A. minisclerotigenes* as *A. flavus* group II. Many researchers have provided evidence that *A. flavus sensu lato* may comprise a variety of species (Geiser et al. 2000; Pildain et al. 2008). Section *Flavi*’s second group of *Aspergillus* includes the nonproducing aflatoxin species *A. oryzae*, *A. sojae*, and *A. tamarii* (Samson et al. 2000).

In crops, there are numerous *Aspergillus* section *Flavi* species that can produce mycotoxins like aflatoxins, cyclopiazonic acid, tenuazonic acid, and 3-nitropropionic acid (Varga et al. 2011). The taxonomy of the aflatoxigenic species in *Aspergillus* section *Flavi* is still unclear, despite numerous publications across a wide range of research fields; several new species (with aflatoxigenic potential) have been described since 2011, including *A. mottae*, *A. transmontanensis*, *A. sergii* (Soares et al. 2012), *A. novoparasiticus* (Gonçalves et al. 2012a, b), *A. bertholletius* (Taniwaki et al. 2012), *A. hancockii* (Pitt et al. 2017), and *A. korhogoensis* (Carvajal-Campos et al. 2017). The precise species identification of strains previously reported as *A. flavus* with big or little sclerotia was also a point of contention (Probst et al. 2014).

In the section *Flavi* of *Aspergillus*, Frisvad et al. (2019) described eight new species: *A. aflatoxiformans*,* A. aspearensis*,* A. austwickii*,* A. cerealis*,* A. neoalliaceus*,* A. pipericola*,* A. subflavus*, and *A. vandermerwei* using a polyphasic approach combining morphology, sequence, physiology, and extrolite data.

The pathway leading to aflatoxin production consists of about 25 genes located in a 70-kilobase DNA region (Yu et al. 2004). The aflatoxin regulatory gene *aflR* in *A. flavus*, *A. parasiticus*, and *A. nidulans* was detected as a transcriptional activator, the three letters “*afl*” are used to indicate the genes of the aflatoxin pathway. Previous studies have shown that *aflA* (*fas-2*, *fas* alpha subunit), *aflB* (*fas-1*, *fas* beta subunit), and *aflC* (*pksA*) are responsible for the conversion of acetate to norsolorinic acid (*nor*) (Brown et al. 1996). Moreover, the uvm8 gene was shown to be essential for *nor* biosynthesis as well as aflatoxin production in *A. parasiticus*. The fatty acid synthase (*fas*) forms the polyketide backbone during aflatoxin synthesis; hence, the uvm8 gene was named *fas-1* (Mahanti et al. 1996). The canonical regulatory genes within the *AF* gene cluster, *aflR* and *aflS*, have been extensively investigated (Zhi et al. 2013). The regulatory gene *aflR* which serves the purpose of stimulating the transcription of genes involved in the process. The *aflR* gene is responsible for encoding a zinc binuclear DNA-binding protein that exhibits sequence-specificity. This protein is a 47-kDa polypeptide of the Gal 4-type and has been demonstrated to play a crucial role in the transcriptional activation of the majority, if not all, of the structural genes. The activation of aflatoxin pathway genes occurs by the binding of the *aflR* protein to the palindromic sequence 5′-TCGN5CGA-3′, which is commonly referred to as the *aflR*-binding motif. This binding event takes place in the promoter region of the structural genes. The motifs that bind to *aflR* are situated within the range of position − 80 to position − 600, with a significant proportion found at positions − 100 to − 200 relative to the translation start site. In certain instances, *aflR* exhibits binding affinity towards an unconventional sequence instead of the motif, as the *aflG* (avnA) gene (Yu et al. 2004). As a diagnostic tool for aflatoxigenic fungi, polymerase chain reaction (PCR) analysis has been used to determine the presence or expression of the aflatoxin biosynthetic gene in some foodstuffs in recent years (Geisen 2007).

There are three possible mechanisms by which fungi act as antagonists in the biological control of other microorganisms and potentially growth-inhibiting: parasitism (using the host’s nutrition), competition (for resources and space), and antibiosis (producing an inhibitory metabolite or antibiotic) (Whipps and Lumsden 1991). While one mechanism is more prevalent, this does not rule out the idea that either of the other two mechanisms, or even both, could also contribute to the antagonistic behavior (Calistru et al. 1997). The *Trichoderma* species are a type of biocontrol agent that has been widely employed as a biopesticide to combat phytopathogenic fungus all over the world (Kaewchai et al. 2009). Mycoparasitism, competition with other fungi for resources and colonization sites, antibiotic production (glyotoxins, viridine, trichodermine, furanone, 6-pentyl-pyrone, and so on), and stimulation of plant defense mechanisms are all ways in which *Trichoderma* species suppress plant pathogens (Liu et al. 2009). The use of *Trichoderma* spores applied directly to seeds is the focus of the majority of biocontrol research. Despite having excellent promise for disease management, *Trichoderma* cannot be applied in the field as a suspension of spores. *Trichoderma* culture should therefore be synthesized as formulations and immobilized in certain carriers for simple application, storage, marketing, and field use (Kumar et al. 2014).

This study aimed to identify and determine the aflatoxin-producing potential of *Aspergillus* section *Flavi* isolates using morphological, molecular, and physiological data and investigate the efficacy of *Trichoderma viride* as a biological control agent in mitigating postharvest infection caused by *A. flavus* in barley and maize grains, with the ultimate goal of reducing aflatoxin levels.

## Materials and methods

### Sample collection

Twenty-three samples of barley (*n* = 15) and maize (*n* = 8) were randomly collected from Qena City retail markets. The samples did not show fungal growth during the collection; they were selected according to the change in the color or texture of the grains. To preserve them until further analysis, we placed the samples in plastic bags and stored them in the refrigerator at 4 °C for 2 h.

### Fungal isolations and identifications

Isolation of fungi was performed on the Czapek Dox agar medium (Oxoid). The agar-plate method was used (ISTA 1999); four grains of each sample were inoculated on the surface of a duplicate culture medium. Five to 7 days were used for incubating all the plates at 28 °C in the dark. Colonies of *Aspergillus* section *Flavi* were transferred for subculturing to Czapek Dox agar medium plates. Macroscopic and microscopic criteria provided by Hedayati et al. (2007), Samson et al. (2014), Frisvad et al. (2019), and Nikolic et al. (2021) were used to perform taxonomic identification of *Aspergillus* section *Flavi* on malt extract agar medium (MEA) (Oxoid), 25 °C for 7 days and for observation of sclerotia, incubation for 20 days. Samson et al. (2014) stated that microscopic observations were obtained from conidiophores grown on MEA after 7–10 days as a standard medium, although other media can also be used when stated in descriptions. We tested Czapek Yeast Agar (CYA) medium for a description of *Aspergillus flavus* species, but no differences were observed.

### Molecular characterization of *Aspergillus* section *Flavi*

#### DNA extraction, amplification, and sequencing

Isolates of *Aspergillus* section *Flavi* were grown for 2 days at 28 °C on potato dextrose agar (PDA) (SRL) medium. Each isolate’s cultured colony was ground in 0.7 ml of 2 × cetyltrimethylammonium bromide buffer (CTAB) (Sigma). All the remaining steps of DNA extraction were performed according to Moller et al. (1992). Electrophoresis on a 1.4% agarose gel, stained with ethidium bromide (Sigma) and visualized under a UV transilluminator, was used to evaluate DNA quality.

A partial *CaM* gene encoding calmodulin was amplified using forward primers CF1M and reverse primer CF4 (Macrogen) (Peterson 2008) (Table 1). The PCR conditions were as follows: initial denaturation at 94 °C for 5 min, followed by 35 cycles of denaturation at 94 °C for 45 s, 55 °C for 45 s, and 72 °C for 1 min, followed by a final extension step at 72 °C for 10 min. Amplification of the internal transcribed spacers of the ribosomal DNA using ITS1/ITS4 primers was performed according to conditions recommended by White et al. (1990).

**Table 1:** List of primers’ names used in this study

Name of primer	Name of the gene	Sequence of primer (5′–3′)	Reference
CF1 CF4	*CaM*	GCCGACTCTTTGACYGARGAR	Peterson et al. (2005)
		TTTYTGCATCATRAGYTGGAC	
AflR1 A	*aflR*	AACCGCATCCTCTCAT	Criseo et al. (2008)
AflR1 B		AGTGCAGTTCAGAACA	
AflR2 A		GCACCCTGTCCTAACA	
AflR2 B		ACGACCATGCCAAGTA	
AflD-1	*aflD (nor-1)*	ACCGCTACGCCGGCAC	
AflD-2		GTTGGCCGCCACTCCG	
AflM-1	*aflM (ver-1)*	AAGTTAATGGCGGAGACG	
AflM-2		TCTACCTGCTCATCGGTGA	

Five microliter amplicon aliquots were electrophoresed with 1.4% agarose gel in TBE buffer (90 mM Tris, 90 mM Boric Acid, 2 mM EDTA, pH 8.3) (Jena Bioscience) after boiling, and ethidium bromide staining and UV transilluminator were performed afterwards. Purifying and sequencing of PCR products were performed at Macrogen (South Korea). The acquired sequences (from one direction) were subjected to BLAST queries against *Aspergillus* section *Flavi* reference sequences from GenBank (https://www.ncbi.nlm. nih.gov/).

#### Phylogenetic analyses

Chromas Lite software was used to edit the sequences that were obtained. We used the CLUSTAL X software in the alignment (Thompson et al. 1994). Phylogenetic analysis was carried out using the obtained sequences as well as additional *Aspergillus* section *Flavi* sequences found in the National Center for Biotechnology Information (NCBI) GenBank nucleotide database (Tamura et al. 2013). A maximum likelihood (ML) method was employed to reconstruct the phylogenetic tree, with bootstrap values obtained after a 1000-run calculation using MEGA software routines. The accession numbers of the studied isolates were indicated in the phylogeny tree.

### Determination of the samples moisture content

By drying a sample at a temperature above the boiling point of water, in an oven with 100–105 °C, until it reached a constant weight (the weight loss is evaluated as a percentage of moisture content), the moisture content of the collected samples was determined (Horwitz and Latimer 2007).

### Detection of the natural occurrence of total aflatoxins and aflatoxin B1 in the samples

A slightly modified immunoaffinity analysis based on the Association of Official Analytical Chemists (AOAC) method was used to quantify total aflatoxins and aflatoxin B1 in the samples (Trucksess et al. 1991). As previously stated by Lewis et al. (2005), the entire sample was powdered, and a 100-g subsample was chosen for analysis. One hundred milliliters of the solvent mixture of methanol and water (80:20) and 5 g of NaCl were added to each sample and mixed quickly in a blender for 3 min. Each sample was then filtered using filter paper (Whatman 2V, Whatman Plc, Middlesex, UK), and the filtrate was then refiltered using glass-fiber filter paper after being diluted with water (1:4). The final filtrate (10 ml) was applied to an AflaTest^®^ WB SR column (VICAM, Watertown, MA, USA) and flowed through it at a rate of 1–2 drops per second. The column was washed twice with 10 ml of water before being eluted with 1 ml of HPLC-grade methanol. The methanol extract was mixed with bromine developer (1 ml), and total aflatoxin or aflatoxin B1 concentrations were measured using a recalibrated VICAM Series-4 Fluorometer set to 360 nm excitation and 450 nm emission. Most of the samples were determined twice to ensure the calibration of the VICAM Series-4 Fluorometer and the used columns.

#### Determination of the total aflatoxin and aflatoxin B1 potentials of *Aspergillus* section *Flavi* isolates

The production of total aflatoxins and aflatoxin B1 was evaluated by culturing the fungal strains in sucrose yeast extract broth medium (SYE), with a composition of sucrose, 40 g; yeast extract, 20 g; and distilled water, 1000 ml, for 15 days at 28 °C, by inoculating an 8 mm disc of 7 days old culture (Ben Fredj et al. 2009). They were extracted using the previously mentioned steps after filtering the fungi and then mixed for 5 min with methanol (100 ml) containing 0.5% NaCl.

#### Detection of aflatoxin biosynthesis genes

For the specific detection of *aflD* (*nor-1*), *aflM* (*ver-1*), and two regions of *aflR* (*aflR1* and *aflR2*), four published primer sets were employed (Criseo et al. 2008). The 400, 537, 798, and 400 bp fragments, respectively, were amplified (Table 1).

PCR was carried out as follows: 5 μl of the master mix (Jena Bioscience) (buffer, dNTP, Taq DNA polymerase, 2 mM Mg Cl_2_), 1 μl of the template DNA, 0.5 μl of both forward and reverse primers, and deionized water to a total volume of 25 μl. The PCR conditions were as follows: 95 °C for 10 min, followed by 30 PCR cycles of 95 °C for 50 s, 58 °C for 50 s, and 72 °C for 2 min, with a final extension step at 72 °C for 5 min (Gallo et al. 2012). PCR products were detected on a 1.4% (wt/vol) agarose gel stained with ethidium bromide.

### Inhibitory activity of *Trichoderma viride* against *A. flavus* during seed storage

#### Fungal strains

The fungal strain of *Trichoderma viride* was obtained from a culture collection in the Applied and Environmental Microbiology Center, isolated from maize seeds, and identified morphologically according to Shah and Afiya (2019). Additionally, the strain of *A. flavus* (F5) was the highest isolate to produce total aflatoxins in this study.

#### Antagonistic effect of *Trichoderma viride* against *A. flavus*

Dual cultures were used to investigate the antagonism and colony contact between *T. viride* and *A. flavus*. A 9-cm-diameter petri dish containing 20 ml of Czapek Dox agar medium was inoculated with an 8-mm-diameter mycelial plug from new cultures of *T. viride* and *A. flavus*, 1 cm apart from the plate edges. The cultures were incubated at 28 °C for 7 days. The formula used to determine the percent inhibition of *A. flavus* radial growth in dual cultures (%I) was (*R* − *R*′)/(*R* × 100), where *R* was the longest radius of the *A. flavus* colony and *R*′ was the radius of the *A. flavus* colony along the line that connected the *A. flavus* and the *Trichoderma* inoculation points (Petchkongkaew et al. 2007).

#### Talc-based formulation

*Trichoderma viride* talc formulation was prepared using the method explained by Jeyarajan et al. (1994). A conical flask was filled with 150 ml of potato dextrose broth medium. Two plates containing a 5-mm, 3-day-old culture of *T. viride* were used as the inoculum, and the medium was incubated for 15 days at 28 °C. The biomass with the medium was mixed into talc at a ratio of 50 ml/100 g of carrier. The mixture was combined with 500 mg carboxy methyl cellulose (CMC)/100 g carrier after being air dried. The contents were sealed in polythene bags and stored in a refrigerator at 4 °C.

#### Seed coating formulation with *T. viride* during storage

After surface disinfection and air drying, 50 g of naturally contaminated barley and maize seeds were coated by hand with 50 g of the formulation of talc powder and *T. viride*. Another talc formulation of *T. viride*-coated seeds inoculated with *A. flavus* was also tested. Seeds inoculated with *A. flavus* or with both *A. flavus* and *Trichoderma viride* were prepared. Seeds free from inoculation were used as controls (naturally contaminated). All the treated and control seeds were incubated at 28 °C for 2 weeks.

## Results

### Mycobiota of different barley and maize samples

The mycobiota of various barley and maize samples marketed in Qena were investigated in this study. All the twenty-three collected samples were contaminated by *Aspergillus* section *Flavi*. One isolate was obtained from each sample. Four, eighteen, and one isolates were identified as *A. aflatoxiformans*, *A. flavus*, and *A. parasiticus*, respectively, by morphological and molecular characteristics (Table 2).

**Table 2:** Morphological characteristics of the studied *Aspergillus* section *Flavi* strains, after incubation at 25 °C for 7 days on malt extract agar medium

	Conidial head				Conidia				Conidiophore wide, μm		Colony					Sclerotia
Isolate no.	Size (wide), μm		Vesicle	Description	Size, μm		Shape	Color			Color and description	Texture	Diameter, mm	Reverse		
***A. aflatoxiformans***																
T16, 28, 32, 35	17.6–35		Subglobose to subclavate	Radiate or loosely columnar (abundant), uniseriate (abundant), and biseriate	2.3–5.6 × 2.6–5.7		Subglobose, smooth	Yellow-green	4.9–10.8		Colonies moderately deep, sulcate; margins entire; mycelium white, abundant, with yellow-green at the edge; sporulation moderately dense; conidia en masse yellow-green and sparse at the margins	Floccose	64–69.5		Buff	Present
***A. flavus* group (1)**																
f4, 9, 13, 17, 22, 25, 44	23.7–44.3		Subglobose to clavate	Radiate and loosely radiate, uni-, and biseriate (abundant)	2.1–6.1 × 1.7–6.5		Subglobose, smooth	Bright green	6.1–13		Colonies moderately deep; margins entire; mycelium green and abundant only in the middle; yellow-green; sporulation moderately dense; conidia en masse bright green	Granulated with floccose in the middle	54.5–70.5		Buff	Present
***A. flavus* group (2)**																
F5, 6, 10, 14, 15, 18, 20, 21, 27, 29, 38	19.1–45.6	Subglobose to clavate		Radiate and loosely radiate, uni-, and biseriate (abundant)		1.8–5.3 × 1.6–5.3	Subglobose, smooth	Bright green to yellow-green	6.7–15.8	Colonies moderately deep, sulcate; margins entire; mycelium white at the margin; green and ochraceous color in the middle colony; sporulation sparse; conidia en masse bright green or yellow-green		Texture floccose, lines may appear at the margin	60–78		Buff	present
***A. parasiticus***																
P24	28.9–36.8	Subglobose to subclavate		Radiate or loosely radiate (abundant), uniseriate (abundant), and biseriate		3.2–5.2 × 2.8–4.5	Subglobose, smooth, dark green	Dark green	11.2–14.9	Colonies moderately deep, sulcate; margins entire; mycelium white at the margin; dense white mycelium of cottony appearance in the middle colony; sporulation sparse; conidia en masse dark green		Granulated with floccose in the middle and border	72		Buff	Present

Moisture content was a significant agent for the contamination of samples with fungi. All samples were found to have a moisture content that ranged from 5.4 to 8.9% (Table 3).

**Table 3:** Code, name, and source of the isolate, moisture content of the sample %, total aflatoxins and aflatoxin B1 ppm, and PCR amplification patterns of aflatoxin genes in the studied *Aspergillus* section *Flavi* population

Strain code	Strain name	Source	Moisture content (%)	Total aflatoxins in the samples (ppm)	Aflatoxin B1 in the samples (ppm)	Total aflatoxins produced by fungi (ppm)	Afla (B) produced by fungi (ppm)	*aflR1*	*aflR2*	*ver* (*aflM*)	*nor* (*aflD*)
T16	*A. aflatoxiformans*	Barley	6.0	0	0	120	11	+	−	−	+
T28	*A. aflatoxiformans*	Maize	7.4	0	0	0	0	−	+	−	−
T32	*A. aflatoxiformans*	Barley	7.8	0	0	0	0	−	−	−	−
T35	*A. aflatoxiformans*	Maize	8.5	0	0	85	8.8	+	+	−	+
f4	*A. flavus*	Barley	5.4	0	0	0.6	0.2	−	+	−	−
f9	*A. flavus*	Barley	5.5	3.4	0	5.3	0.8	+	+	+	+
f13	*A. flavus*	Barley	5.6	0	0	0.1	0	−	+	+	+
f17	*A. flavus*	Barley	5.6	0	0	0.3	0	−	−	−	−
f22	*A. flavus*	Maize	6.3	76	9.5	0	0	−	−	−	−
f25	*A. flavus*	Barley	6.2	0	0	0	0	−	−	+	−
f44	*A. flavus*	Maize	7.3	0	0	2.2	1.1	+	−	−	−
F5	*A. flavus*	Barley	5.8	0	0	240	8.6	−	−	−	−
F6	*A. flavus*	Barley	5.4	2.1	0.5	93	4.9	+	−	−	−
F10	*A. flavus*	Barley	6.0	0	0	3.9	3.1	+	−	−	+
F14	*A. flavus*	Maize	5.4	0	0	31	4.7	+	+	+	+
F15	*A. flavus*	Barley	6.1	0	0	120	6.6	+	+	−	−
F18	*A. flavus*	Barley	6.1	0.7	0.2	92	4.8	+	+	+	+
F21	*A. flavus*	Barley	5.6	0	0	0	0	−	−	−	−
F20	*A. flavus*	Maize	6.4	64	12	0	0	−	+	+	+
F27	*A. flavus*	Maize	6.9	0	0	0	0	+	+	+	+
F29	*A. flavus*	Barley	5.9	0	0	2.8	1.2	+	+	−	−
F38	*A. flavus*	Maize	8.9	0	0	0	0	−	−	+	+
P24	*A. parasiticus*	Barley	6.4	0	0	2.4	1.6	+	+	+	−

### Morphology of the *A. flavus* species complex

**Fig. 1:**
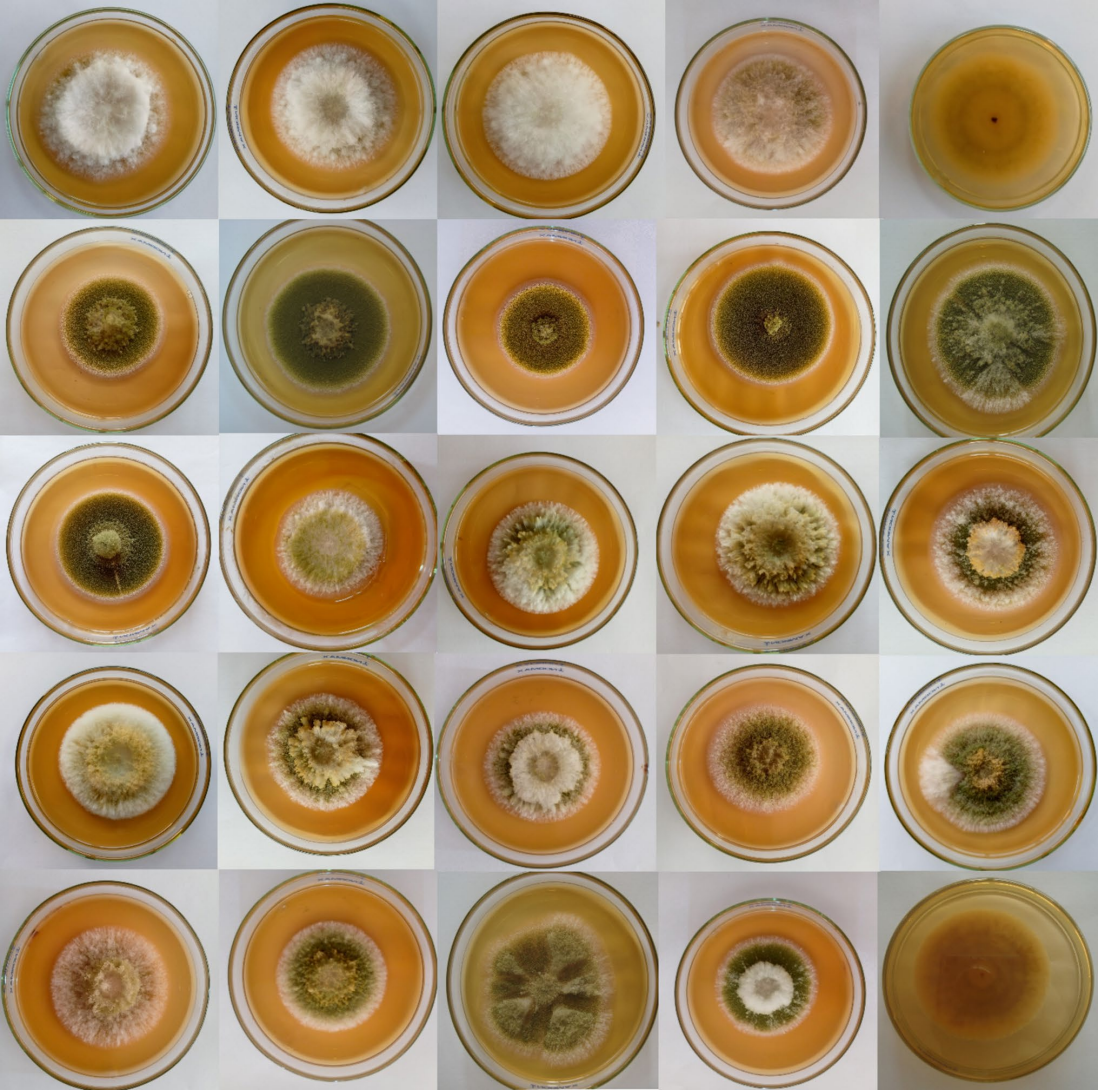
7 days old colonies on MEA at 25 °C: left to right 1–4: *A. aflatoxiformans*, 5: reverse colony of *A. aflatoxiformans*, 6–11: *A. flavus* group (1), 12–23: *A. flavus* group (2), 24: *A. parasiticus*, 25: the reverse of *A. flavus* colony

**Fig. 2:**
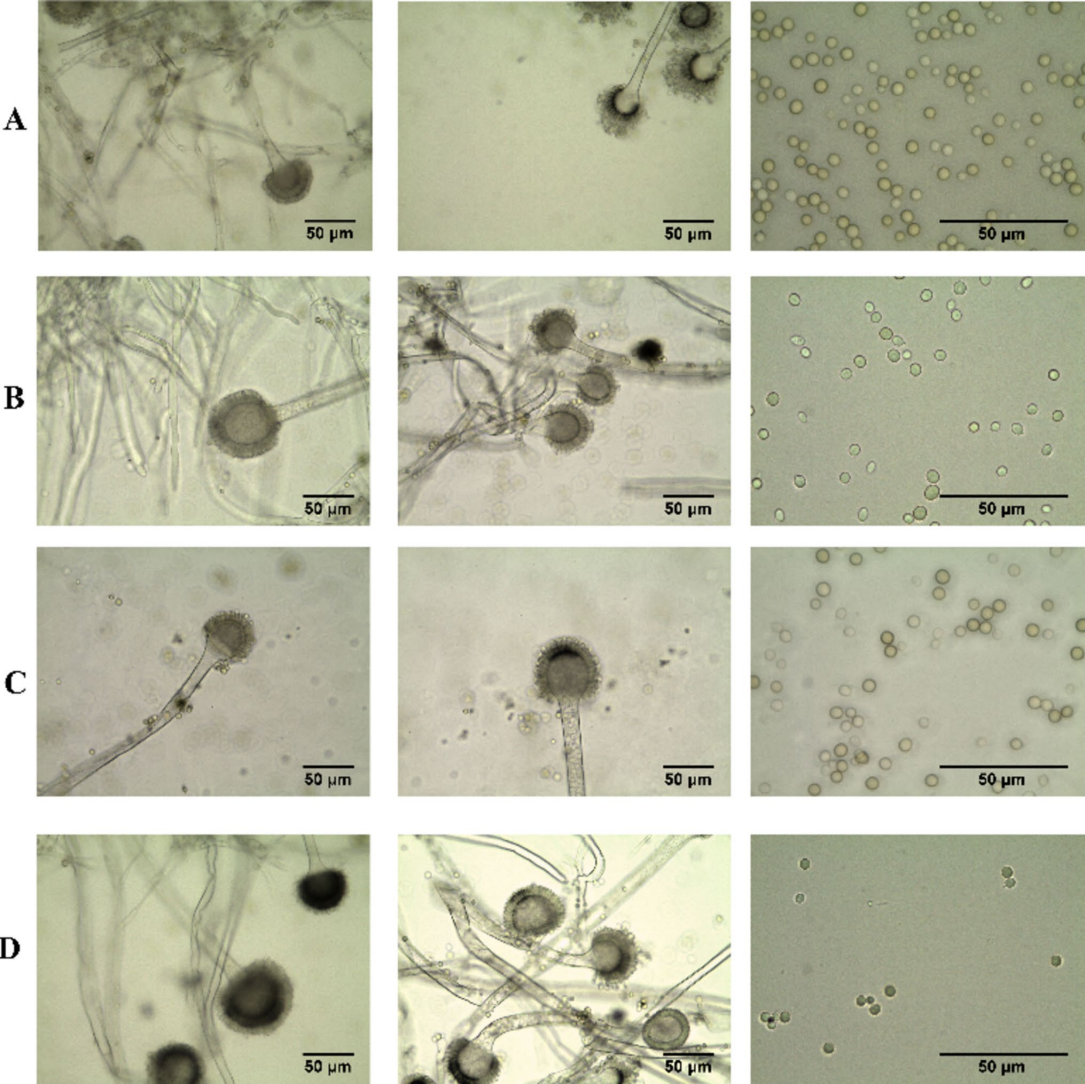
Microscopic characteristics after a 7-day incubation period on MEA at 25 °C, *Aspergillus aflatoxiformans* (**A**), *A. flavus* (**B**, **C**), and *A. parasiticus* (**D**). From left to right: 1, 2, 4, 5, 7, 8, 10, and 11 at × 40 and 3, 6, 9, and 12 at × 100

**Fig. 3:**
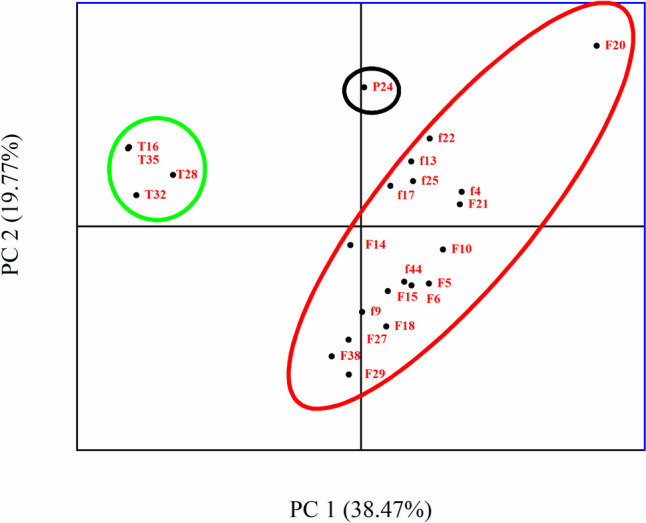
Principal component analysis (PCA), analysis of *Aspergillus* section *Flavi* based on the morphology, isolates inside the red circle was *Aspergillus flavus* groups; the green circle was *A. aflatoxiformans*, and the black circle was *A. parasiticus*

On MEA medium at 25 °C for 7 days, *A. aflatoxiformans* appeared as follows: colonies moderately deep, sulcate; margins entire; mycelium white, abundant, slightly yellow-green color sometimes found at the edge; sporulation moderately dense; conidia en masse yellow-green and sometimes slightly sparse at the margins. The colony texture was floccose, with a diameter ranging from 64 to 69.5 mm and a buff color on the reverse. Sclerotia had a black appearance. The conidial head was radiate or loosely columnar (abundant), uniseriate (abundant), and biseriate. The vesicle was subglobose to subclavate, with a width of 17.6–35.0 μm. The conidiophore width was 4.9–10.8 μm. Conidia were smooth and subglobose, with a diameter of 2.3–5.6 × 2.6–5.7 μm (Table 2, Figs. 1, 2, and 3).

*A. flavus* was divided into two groups according to the colonies. The first group showed colonies moderately deep; margins entire; mycelium green and abundant only in the middle; yellow-green; sporulation moderately dense and bright green conidia en masse. The texture of the colony is granulated and floccose in the middle with a diameter of 54.5–78 mm; reverse with a buff color. Sclerotia appeared black. The conidial head was radiate and loosely radiate, uni-, and biseriate (abundant). Vesicle morphology ranged from subglobose to clavate, with a width of 23.7–44.3 μm. The conidiophore width was 6.1–13.0 μm. Conidia were subglobose and smooth; with a bright green color and a diameter of 2.1–6.1 × 1.7–6.5 μm (Table 2, Figs. 1, 2, and 3).

The second group in *A. flavus* exhibited colonies moderately deep, sulcate, with entire margins; mycelium was white at the margin; green and ochraceous color in the middle of the colony; sporulation sparse; conidia en masse bright green. The colony showed a floccose texture; the reverse was buff in color. Sclerotia were black in color. The conidial head was radiate and loosely radiate, uni-, and biseriate (abundant), sometimes loosely columnar was abundant. The vesicle was subglobose to clavate, with a width of 19.1–45.6 μm. The conidiophore width was 6.7–15.8 μm. Conidia were subglobose, smooth, bright green in color, and with a diameter of 1.8–5.3 × 1.6–5.3 μm (Table 2, Figs. 1, 2, and 3). F20 isolate was distinguished by the appearance of uniseriate conidial head, and colony texture granulated with floccose in the middle and extend to the margin in lines. Principal component analysis (PCA) in Fig. 3 analyzed the obtained characteristics; the isolates of two groups of *A. flavus* were grouped in the same population, and although F20 showed a slight distance from others, it was identified as *A. flavus* after molecular identification (it showed low similarity by *CaM* gene 71% and confirmed with ITS region that showed 100% similarity to *A. flavus*).

The *A. parasiticus* colony was moderately deep and sulcate, with entire margins and white mycelium at the margins; dense white mycelium with a cottony appearance in the middle of the colony; sporulation was sparse; conidia en masse dark green. The texture was granulated with floccose in the middle and border. The colony had a diameter of 60.5 mm; the reverse was buff in color and sclerotia appeared. The conidial head was radiated or loosely radiated (abundant), uniseriate (abundant), and biseriate. The vesicle was subglobose to subclavate with a width of 28.9–36.8 μm. Conidia were subglobose, smooth, and dark green and had a diameter of 3.2–5.2 × 2.8–4.5 μm. The conidiophore width was 11.2–14.9 μm (Table 2, Figs. 1, 2, and 3).

### Phylogeny of the *A. flavus* species complex

**Fig. 4:**
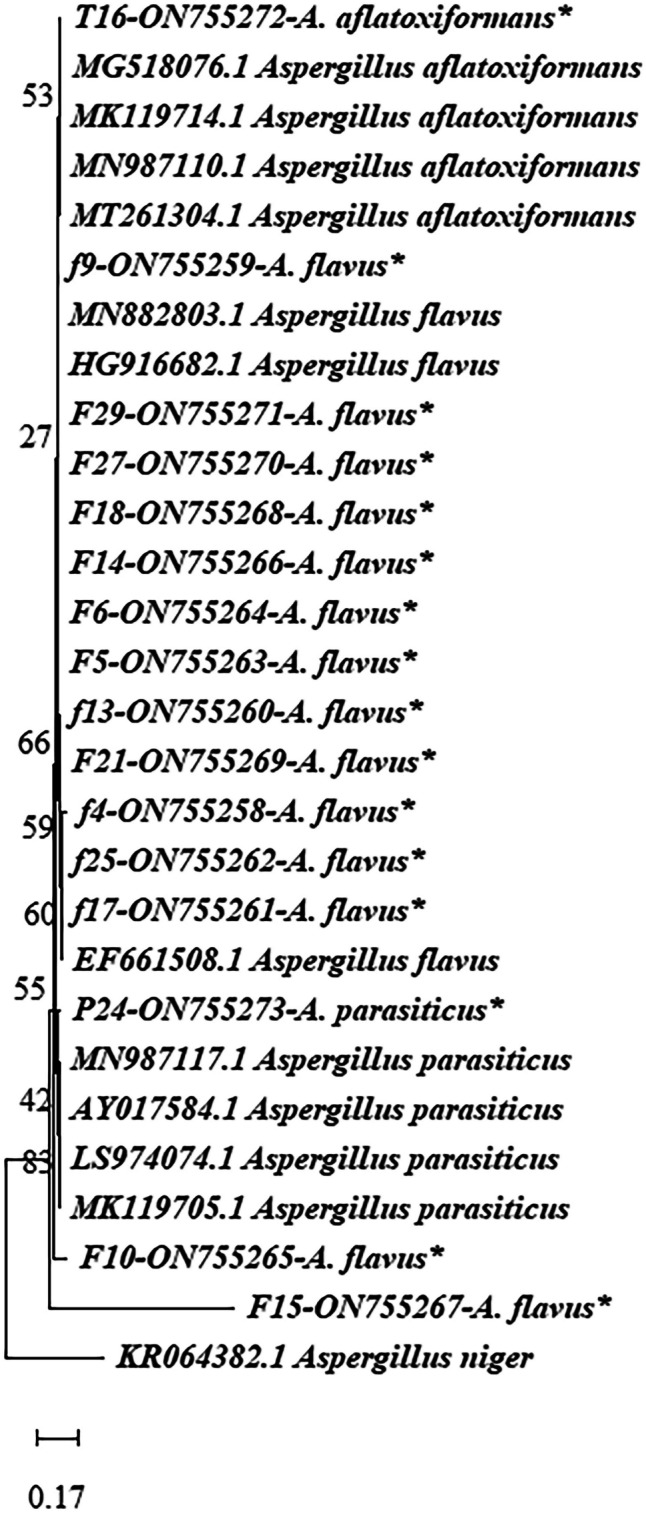
Phylogenetic tree of *Aspergillus* section *Flavi* strains isolated from barley and maize based on calmodulin sequence data. The numbers above branches indicate bootstrap values that were constructed after a run of 1000 replications (isolates of this study were marked with stars)

The BlAST results of sequences from the *A. flavus* species complex showed 92–100% similarity to *A. flavus* species deposited in GenBank. Twenty-three isolates were successfully sequenced, and sixteen out of them were shown in the phylogenetic tree, the other seven isolates were identified based on morphology because they showed low similarity. F20 isolate sequenced with ITS region to confirm the identification that exhibited slight morphological differences from other identified isolates and showed 100% *A. flavus*. The accession numbers were shown in the phylogenetic tree; it revealed that the isolates could be categorized into three clades, each representing one species (Fig. 4).

Fourteen strains (5, 29, 18, 9, 14, 6, 13, 21, 17, 25, 27, 10, 4, and 15) were clustered in the same clade with *A. flavus* obtained from GenBank with accession numbers MN882803.1 and HG916682.1. *A. parasiticus* strain no. 24 was grouped with the accession number MN987117.1 and LS974074.1 with a bootstrap value of 99%. *A. aflatoxiformans* strain no. 16 was arranged in the same clade containing *A. aflatoxiformans* with accession number MN987110.1 and MT261304.1 obtained from GenBank (Fig. 4).

### Natural occurrence of aflatoxins in barley and maize samples

Five samples of barley and four of maize out of twenty-three tested samples were naturally contaminated with total aflatoxins and aflatoxin B1. The contamination fluctuated from 0.7 to 64 and 0.2 to 12 ppm, respectively. Five samples were contaminated with aflatoxins, and 18 samples were free from aflatoxins (Table 3).

### Aflatoxin production by isolates of *Aspergillus* section *Flavi*

Twenty-three isolates of *A.* section *Flavi* were screened for total aflatoxin and aflatoxin B1 potential. Total aflatoxins and aflatoxin B1 production varied by tested strains. Fifteen isolates were detected to produce total aflatoxins. The total aflatoxin potential ranged between 0.1 and 240 ppm. F5, isolated from barley, exhibited the highest value of total aflatoxins. Thirteen isolates showed aflatoxin B1 potentials with different levels ranging from 0.2 to 8.8 ppm. T35 produced the highest level of aflatoxin B1 (Table 3).

### PCR patterns of aflatoxin biosynthetic genes

In this study, primer pairs were used to target three aflatoxin biosynthetic genes: two regions in the regulatory gene *aflR* and the structural genes *aflD* and *aflM*.

The twenty-three isolates were tested for the amplification of the three aflatoxin biosynthetic genes. All four genes were amplified from four strains, as shown in Table 3. Three of these strains (f9, F14, and F18) were able to produce total aflatoxins and aflatoxin B1, and the fourth, isolate of F27, failed to produce total aflatoxins and aflatoxin B1. Additionally, the two regions of regulatory genes *aflR1* and *aflR2* were amplified in four isolates (T35, F15, P24, and F29), which exhibited total aflatoxin and aflatoxin B1 potentials. Additionally, *aflR2* was amplified from four strains, T28, f4, f13, and F20; of these, f4 could produce total aflatoxins and aflatoxin B1, f13 produced total aflatoxins only, and the other strains were unable to produce any toxins. *AflM* was amplified from f25, P24, and F38*. AflD* could not be amplified from six isolates (T16, T35, f13, F10, F20, and F38) (Table 3).

### Inhibitory effect of *T. viride* on the growth and total aflatoxin production of *A. flavus*

**Fig. 5:**
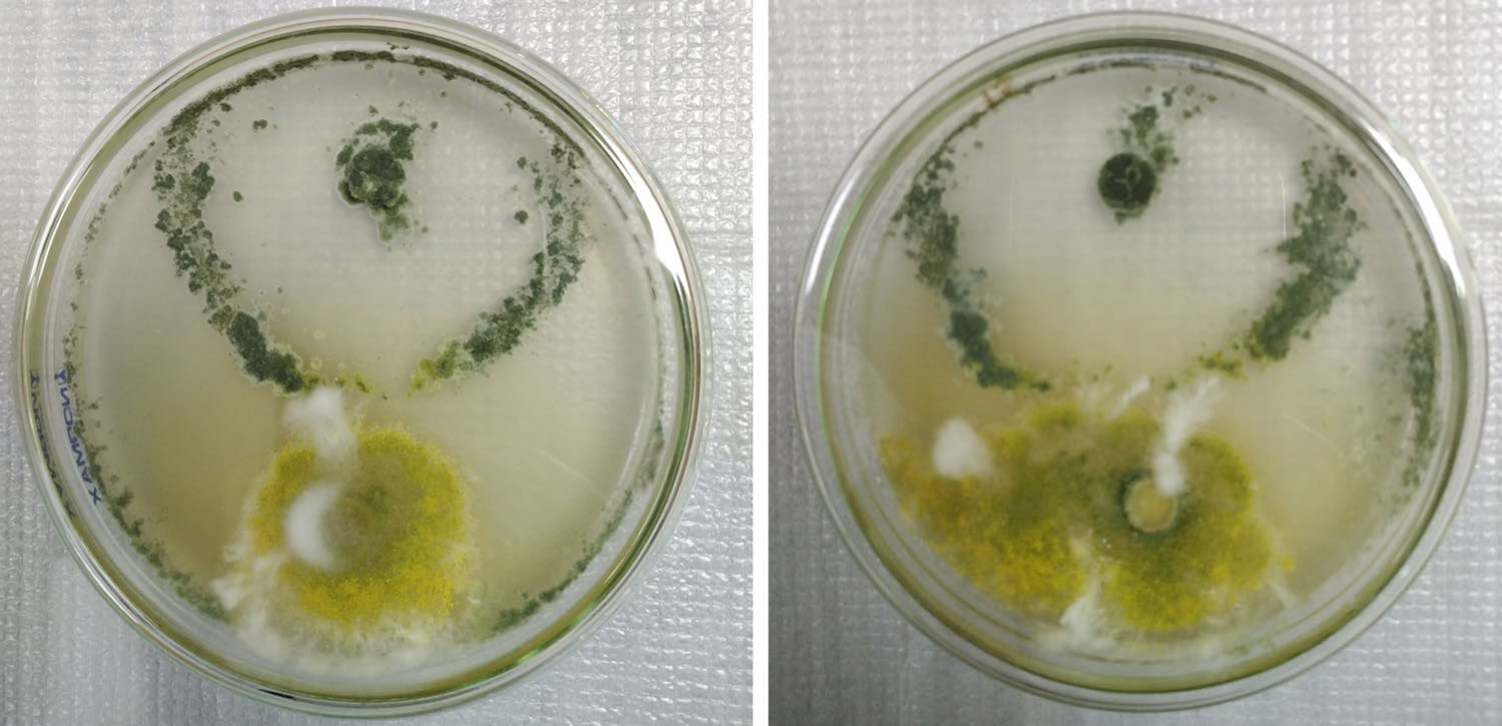
The growth inhibition effect of *Trichoderma viride* on *Aspergillus flavus* on Czapek Dox agar medium at 28 °C for 7 days

The inhibitory effect of *T. viride* on *A. flavus* was investigated in the Czapek Dox agar medium. For the *T. viride* tested, the growth inhibition effect of *A. flavus* was 31.3% (Fig. 5).

The inhibitory effect of *T. viride* on total aflatoxin was investigated using naturally contaminated barley and maize seeds, stored at 28 °C for 15 days. Total aflatoxins were found in untreated seeds (controls, naturally contaminated) at 46 and 43 ppm in barley and maize, respectively. Total aflatoxin production by *A. flavus* in inoculated seeds was 200 and 240 ppm. The inhibitory effect of *T. viride* on total aflatoxin production by *A. flavus* in inoculated seeds was 59 and 95 ppm, respectively. The inhibitory effect of *T. viride* on total aflatoxins in untreated and formulated seeds was 8.6 and 2.1 ppm, respectively, as compared with control. Total aflatoxins were significantly lower in seeds treated and formulated (with *A. flavus* and *Trichoderma viride*), i.e., the production of total aflatoxins by *A. flavus* was lower in the seeds formulated by *T. viride* with 2.8 and 1.3 ppm (Table 4).

**Table 4:** Total aflatoxins in the stored samples and antagonistic effect between *Trichoderma viride* and *Aspergillus flavus*

Treatment type	Aflatoxins in maize seeds (ppm)	Aflatoxins in barley seeds (ppm)	Mycelial inhibition (%)
C	43	46	
A	240	200	
A + T	95	59	31.3
T + F	2.1	8.6	
A + T + F	1.3	2.8	

*C* control, *A* *A. flavus,* *T* *Trichoderma viride,* *F* formulation

## Discussion

In this study, *Aspergillus* section *Flavi* was the dominant group isolated from all collected barley and maize samples; also, other fungi were recorded as rare. This study focused on the description of the *Aspergillus flavus* group because it was the predominant section. According to reports from maize-growing regions all over the world, *Aspergillus* species belonging to the section *Flavi* were the most common aflatoxigenic species in maize (Horn 2007; Gallo et al. 2012). Both *A. flavus* and *A. niger* were able to frequently and extensively infect grain from a variety of industrial crops (soybean and sunflower) and cereals (barley, maize, and wheat) in Serbia under certain agroecological circumstances (Levic et al. 2012). Hadiani et al. (2009) found that the incidences of aflatoxin B1, aflatoxin B2, and total aflatoxin in maize samples were 66, 54, and 63%, with mean values of 9.5 ± 16.3, 1.7 ± 2.6, and 10.4 ± 18.4 ng/g, respectively.

The obtained morphological characteristics of *A. aflatoxiformans* were partially similar to those described by Frisvad et al. (2019). A biseriate conidial head appeared in our results, vesicle size was 17.6–35 μm, and conidia size was in the range 2.3–5.6 × 2.6–5.7 μm. In contrast to Frisvad et al. (2019), who found only a uniseriate conidial head, conidiophores appeared with rough stipes, vesicle wide reached to 38 μm, and conidia were 3.5–5 × 3–4.5 μm.

Two different colony morphologies and surface textures were observed for *A. flavus* in this investigation. In the first group, colonies appeared to be green mycelium abundant only in the middle and granulated with a floccose texture also in the middle. In the second group, colonies were sulcate, mycelium was white at the margins, green and ochraceous color in the middle of the colony, and floccose texture. Vesicle diameter was 19.1–45.6 μm, and conidia size was 1.8–6.1 × 1.6–6.5 μm, and smooth. In contrast, Hedayati et al. (2007) reported that conidiophores were roughened and heavy-walled. Vesicles showed diameters ranging from 10 to 65 μm. Conidia fluctuated between 3.5 and 4.5 μm in diameter, and echinulate.

In this research, *A. parasitcus* phialides appeared as uniseriate (abundant) and biseriate, vesicle size was 28.9–36.8 μm, and conidia size reached to 2.8–5.2 μm, smooth and dark-green. This finding was in contrast to Hedayati et al. (2007), who found that phialides were in one series, and vesicles were 20–35 μm in diameter. Conidia were coarsely echinulate, 3.5–5.5 μm in diameter, and bright yellow-green.

All isolates of *Aspergillus* section *Flavi* produced black sclerotia in this study; this result was in agreement with Houbraken et al. (2020).

The calmodulin gene sequencing results indicated that the relationship between species of *A. flavus* was close. The calmodulin gene was chosen for its accurate discrimination of species than other genes. In a study carried out by Samson et al. (2014) on the nomenclature of *Aspergillus*, they reported that the RPB2 gene is not easy to amplify, rendering its use as a secondary identification marker frustrating. In contrast, BenA is easy to amplify, but has been reported to vary in the number of introns and PCR sometimes results in the amplification of paralogous genes (Peterson 2008). Otherwise, *CaM* is easy to amplify and distinguishes among all *Aspergilli*. In addition, the *CaM* sequence database is almost complete for all accepted species. As such, from a practical point of view, they suggest the use of *CaM* as a temporary secondary identification marker in *Aspergilli*. Okoth et al. (2018) revealed that the analysis of calmodulin gene sequences showed a greater degree of variance across the *A. flavus* isolates, with certain isolates showing closer similarity to *A. minisclerotigenes* than to others based on β-tubulin gene sequences. However, no significant variation was observed in the ITS sequences. The results of Alshehri and Palanisamy (2020) revealed that the targeting of the calmodulin gene yielded superior resolution in comparison to the ITS region and β-tubulin gene. Specifically, the classification of aspergilli in the *Flavi* section based on clades showed that calmodulin sequences provided more resolution compared to the others. Based on the results of Frisvad et al. (2019), it was found that the *A. flavus* clade contained 15 species, including *A. aflatoxiformans*, *A. flavus*, and *A. parasiticus*, and they represented the majority of species in section *Flavi*. This was determined by using maximum likelihood analysis of three merged gene sequences (*BenA*, *CaM*, and ITS rDNA region) in the phylogenetic tree. According to β-tubulin (*BenA*) and calmodulin (*CaM*) partial genes sequencing analysis, Gallo et al. (2012) identified all 67 of their isolates as *A. flavus*. All 67 tested strains were found to cluster together with the *A. flavus* type strain (ITEM 7526), according to the evolutionary tree reconstructed using the neighbor-joining method (with a high bootstrap value).

In this study, seven isolates were identified by morphological features and we could not identify them by *CaM* gene because their DNAs were amplified successfully and sequenced, but they did not show similarity or exhibited low similarity (71%) with deposited sequences found in the GenBank when subjected to blasting. One isolate from them was subjected to ITS sequence as slightly different from others, and it showed similarity 100% to *A. flavus*. Morphology and molecular identification need more efforts to develop the accurate identification of *Aspergillus* section *Flavi*, because it is a complex group containing various species, they showed similarity in morphology and molecular characteristics. In a study carried out by Saber et al. (2022), they reported that the four diagnostic techniques—phenotyping, PCR, PCR–RFLP, and amplicon sequencing—have demonstrated varying degrees of sensitivity in characterizing of *Aspergillus flavus* group. Phenotypic tools were restricted to the identification of *A.* section *Flavi*; PCR by itself was unable to identify *A*. *parasiticus* from *A. flavus*, also in their study, PCR–RFLP was only able to detect *A. flavus*. It was unable to differentiate between various *A. flavus* strains, though. The several *A. flavus* strains might be distinguished using sequencing and sequence analysis. Based on the degree of fungus strain segregation, these data determined the criteria for method selection. For the detection of the pathogenic *A. flavus* in grain crops, new techniques based on Raman spectrometry to assess AF-B1 alone or in conjunction with other fungal components may be helpful.

In this study, we screened *A.* section *Flavi* strains, including aflatoxigenic and nonaflatoxigenic strains, for the presence or absence of four aflatoxin genes. All aflatoxin- and nonaflatoxin-producing isolates showed the entire set of genes or only three, two, one, or none. All strains producing aflatoxins were linked to the presence of *aflR1* and/or *aflR2*, except two isolates that exhibited aflatoxins, but from which *aflR1* or *aflR*2 could not be amplified. Two regulatory genes, *aflR* and *aflS*, and five structural genes, *aflD*, *aflM*, *aflO*, *aflP*, and *aflQ*, were reported by Gallo et al. (2012). Three strains were not aflatoxin producers or produced aflatoxins in amounts below the minimal detectable value, despite demonstrating all seven amplification products, i.e., *aflR*, *aflS*, *aflQ*, *aflP, aflD*, *aflM*, and *aflO*. In these three strains, one or more of the additional genes involved in aflatoxin production are likely missing or have deletions (Gallo et al. 2012).

Our study detected one regulatory gene *aflR*; the isolates showed aflatoxin production in the absence of it. They may have an *aflS* gene that is responsible for the production and was not studied here, or the DNA nucleotides were subjected to the mutation and the primer missed the genetic site. According to Geisen (1996), the distinction between aflatoxigenic and nonaflatoxigenic strains of *Aspergillus flavus* may also arise through straightforward genetic alterations, such as nucleotide substitutions. Gherbawy et al. (2015) found that three isolates of *Aspergillus tamari* failed to amplify any DNA pattern, *aflR*, *nor-1*, *ver-1*, and *omtA*. In contrast, Klich et al. (2000) observed that the DNAs of *A. tamarii*, *A. flavus*, and *A. parasiticus* exhibited amplification of the four genes, suggesting significant resemblances in the genes associated with the biosynthetic pathway among these three species. In reality, regulatory genes, alterations, environmental variables, and so on, all have a role in controlling aflatoxin production, in addition to the structural genes in the cluster (Li and He 2018). Aflatoxin synthesis in *A. flavus* can also be influenced by a number of other regulatory factors (Lin et al. 2013; Zhao et al. 2017, 2018), including LaeA, Ham, NosA, FarB, and CreA.

Twenty-five genes, located within a 70-kb DNA sequence on the chromosome, regulate the formation of aflatoxin B1, and their DNA sequences have been reported (Criseo et al. 2001; Yu et al. 2004; Scherm et al. 2005). The norsolorinic acid reductase-encoding gene *nor-1*, the versicolorin A dehydrogenase-encoding gene *ver-1*, the sterigmatocystin 0-methyltransferase-encoding gene *omt-1*, and the regulatory gene *aflR* were used in PCR for the screening of aflatoxigenic aspergilli (Erami et al. 2007).

According to research conducted by Chang et al. (2005), loss of aflatoxin production capacity was associated with mutations or deletions of genes. Moreover, Criseo et al. (2008) discovered that nonaflatoxigenic *A. flavus* strains exhibited distinct DNA banding patterns and were lacking between one and four genes (*aflM*, *aflP*, *aflR*, and *aflD*). Nonaflatoxigenicity could be caused by the complete loss of a gene, a part of or the entire biosynthetic cluster, or alterations at the primer binding sites, according to these studies. These uncertainties highlight the challenges of biosynthetic gene amplification for aflatoxin production diagnostics.

Seed contamination with aflatoxins is a significant problem for global trade and food safety. Controlling food chain contamination at several stages, including agriculture, distribution, storage, and processing, is essential (Torres et al. 2014). It was observed in this study that *Trichoderma viride* exhibited inhibition of total aflatoxins in untreated (control) and formulated seeds at 8.6 and 2.1 ppm, respectively, compared with control seeds, which showed total aflatoxins at 46 and 43 ppm in barley and maize seeds, respectively. *T. harzianum* in a talc-based powder formulation remained viable for 180 days at temperatures ranging from 0 to 40 °C, according to Gaur et al. (2005).

In this study, *Trichoderma viride* inhibited the growth of *A. flavus* by 31.3%. Ren et al. (2022) reported that the biological control of *Trichoderma* strains may inhibit the growth of fungi, decrease the production of aflatoxin, or both. While acting in a variety of ways, *Trichoderma* spp. metabolites contribute to their mechanism of action. Some prevent the growth of *A. flavus*, while others, possibly through degradation, minimize the production of aflatoxins.

In conclusion, the subgroup distinctions revealed in the *A. flavus* population require additional investigation to determine whether they reflect reproductively isolated subgroups. The selection of safe and effective toxigenic strains for the biological control of aflatoxins may be aided by molecular characterization focused on biosynthetic genes. In this study, using *Trichoderma viride* to biocontrol *A. flavus* growth and aflatoxins during seed storage was recommended.

## Data Availability

All data generated or analyzed during this study were included in this manuscript.

## Abbreviations

afl: Aflatoxin genes pathway
*CaM*: Gene encoding calmodulin
CMC: Carboxymethyl cellulose
CTAB: Cetyltrimethylammonium bromide buffer
fas: The fatty acid synthase
MEA: Malt extract agar media
NCBI: National Center for Biotechnology Information
nor: Norsolorinic acid
PDA: Potato dextrose agar medium
SYE: Yeast extract broth medium
